# Patterns in refractive error and treatment delay in keratoconus–An Australian study

**DOI:** 10.1371/journal.pone.0297268

**Published:** 2024-01-11

**Authors:** Samantha Bobba, Alanna Wood, John Males, Yves Kerdraon

**Affiliations:** 1 Department of Ophthalmology, Westmead Hospital, Sydney, New South Wales, Australia; 2 Department of Ophthalmology, Sydney Eye Hospital, Sydney, New South Wales, Australia; 3 Department of Ophthalmology, Save Sight Institute, University of Sydney, Sydney, New South Wales, Australia; Save Sight Institute, AUSTRALIA

## Abstract

Keratoconus is the most common primary corneal ectasia and is associated with significant morbidity. In its early stages, keratoconus is often asymptomatic, making the identification of subclinical disease challenging. Refractive error is a parameter that is documented at most routine optometry visits, yet interestingly, changes in refraction of keratoconic patients over time have not yet been studied and compared with the general population. Early diagnosis of keratoconus facilitates timely referral for treatments such as corneal collagen cross-linking, which has been shown to slow disease progression. In this context, documenting delays between initial presentation to the optometrist and referral for collagen-cross-linking as well as comparing the trends in visual acuity and refractive error between keratoconic and non-keratoconic patients over time are particularly relevant.

## Introduction

Keratoconus is the most common primary corneal ectasia and is associated with significant ocular morbidity due to its typically progressive nature and relatively young age of onset, commonly presenting in the teenage years to twenties [1, 2]. Keratoconus often remains asymptomatic in its early stages, with visual decline and irregular astigmatism usually manifesting later in the course of the disease [3, 4]. Studies following from the Save Sight Keratoconus Registry (an Australian-based database that collects real-world data involving optometrists and ophthalmologists before and after corneal collagen cross-linking [CXL]) report a steeper Kmax and younger age as the most clinically useful baseline predictors of keratoconic progression. Each year younger was associated with a 4% and 2% greater risk of steepening Kmax and thinnest corneal thickness, respectively, emphasising the need for early detection of keratoconus in reducing disease morbidity [5]. Screening and diagnostic modalities have continued to evolve, with a range of topographic and tomographic parameters frequently utilised by ophthalmologists in diagnosis and grading of keratoconus [6–9]. A consensus on the gold standard approach to diagnosis and classification of keratoconus and disease progression is yet to be established [2, 9, 10].

Early keratoconus, in particular, is difficult to detect without advanced imaging modalities such as topography and tomography, making the detection of disease particularly difficult for optometrists, who are most likely to encounter asymptomatic patients with subclinical keratoconus in their routine practice, but commonly either do not have access to advanced imaging modalities or do not utilise them in screening the general population [11–13]. A recent survey of optometrists in New Zealand identified a significant discrepancy in specialist referral criteria for patients with suspected keratoconus, with 41% reporting referral on progression of corneal parameters, 21% at no set time and 27% on initial diagnosis [13]. This is concerning given that keratoconus may remain undetected for years in its early stages, with the lack of consistency in referral likely contributing to delays in the ability of ophthalmologists to offer corneal collagen cross-linking as a potential therapy. Another New Zealand-based prospective observational study of 96 patients with keratoconus at a tertiary centre found that 39.6% had significant disease progression over a period of 153 days whilst waiting for CXL, and suggested that a risk stratification score may help reduce progression [14]. Unfortunately, there are currently no guidelines from the Royal Australian and New Zealand College of Ophthalmologists regarding when to refer keratoconic patients either for ophthalmology review or to corneal subspecialists for CXL therapy. This is not unique to Australia and New Zealand, with a recent retrospective review in Belgium also reporting that keratoconus is often diagnosed late in the course of the disease with delays in subspecialist referral, and advocating for research into screening strategies [15]. On a similar note, a survey of ophthalmologists in the United Kingdom reported variations in the monitoring of corneal ectasia and indications for CXL [16], indicating a need for a global consensus not only in referral guidelines but also regarding keratoconic progression and management.

Recent literature has considered the role of machine learning techniques and neural networks in detecting subclinical keratoconus, increasingly relevant in today’s era of increased access and availability of refractive surgery [17–21]. A key criticism of neural networks in detecting keratoconus however, is that their use has been limited to further analysing data from expensive topographers or aberrometers, reducing their viability as a screening method. Indeed, further studies are required before such neural network approaches could become a mainstream screening tool [10, 22].

Refraction and visual acuity are important yet often undervalued tools in the work-up and monitoring of keratoconus progression. Of note, the Pentacam’s ABCD progression display utilises distance visual acuity as one of the indices to document keratoconus progression, however, most studies focus on the role of the former 3 ABC parameters in detecting disease progression with less emphasis on the role of visual acuity in the literature [7, 23, 24]. To date, no study has specifically investigated rates of progression in best spectacle-corrected visual acuity (BSCVA) or refractive error in keratoconic patients prior to diagnosis, nor compared these parameters between keratoconic and non-keratoconic subjects. Although retinoscopy and autorefractors are often used in optometry and can be helpful in keratoconus detection [25], evaluating trends in BSCVA and subjective refraction is of particular importance, given that they are more commonly utilised tools by optometrists in screening the general adult population. In a longitudinal study of 34 eyes, Shirayama-Suzuki and colleagues (2009) showed that progression to keratoconus was correlated with more asymmetry of corneal power and regular astigmatism at the initial presentation, suggesting that astigmatism could be another factor to consider in the clinical diagnosis of progressive keratoconus [26]. More recently, Greenstein and colleagues (2023) retrospectively reviewed the subjective refraction (amongst other parameters) of keratoconic patients in a single-centre corneal and refractive surgery practice over a ten-year period. They reported a mean subjective sphere of -2.2D and cylinder of -3.2D, with 48.6% of patients having against the rule astigmatism, suggesting that refractive data can aid clinicians in flagging suspect patients that would benefit from further investigations such as corneal tomography [27]. Their study did not, however, review refractive data from keratoconic patients’ referring optometrists, and thus is limited to identifying patterns in refractive error in patients with already established disease, rather than in its early stages which is more relevant to disease screening and facilitating early detection of keratoconus. To the authors’ knowledge, our study is the first to document patterns in BSCVA and subjective refraction cylinder progression in keratoconic patients that have undergone CXL with optometry-based refractive data from prior to their diagnosis. The study also analyses delays from first optometric presentation to the crosslinking procedure, and provides a comparison with refractive data in non-keratoconic patients, which has previously not been reported in the literature.

## Materials and methods

Patients with keratoconus that had undergone CXL were recruited from three private ophthalmic practices across New South Wales (NSW), Australia between 2016–2017. Referring optometrists were contacted by the authors with informed written consent from patients (and their guardians/parents in the case of minors) to determine the first recorded date at which they had reviewed the patients, recorded measurements of BSCVA, subjective refraction over time (four to six visits recorded for each patient), and the date at which the keratoconic patients were referred to an ophthalmologist. The dates of CXL and comorbid ocular and systemic disease were obtained from the ophthalmic practices. The criteria for keratoconic progression and when to perform CXL combined progression in Kmax, K2, posterior float, worsening BSCVA and a clinical assessment of risk of future progression that included age, active allergic eye disease and persistent eye-rubbing. The data was de-identified and recorded in a password-protected database, after which point authors did not have access to information that could identify individual participants.

BSCVA (visual acuity) data was collected simultaneously for a control group of subjects without documented evidence of corneal ectasia or prior refractive surgery from two optometry practices in Wollongong, New South Wales. A conversion from Snellen to logMAR BSCVA was used to allow for comparison between practices and statistical analysis. In order to age-match both patient groups given the typical age of onset of keratoconus, patients in the control group were included only if they were aged 35 years or younger. Collection of visual acuity and refractive data was approved by the local ethics committee (South Eastern Sydney Local Health District Ethics Committee, HREC 17/137, refer to S2 File “Ethics Committee Approval”) and conducted in accordance with the tenants of the Declaration of Helsinki. The need for consent of the control group was waived by the ethics committee given the de-identified nature of records and retrospective data collection. Authors did not have access to information that could identify individual participants in the control group during or after data collection.

Demographic data, comorbid ocular and systemic disease, visual acuities and refractive data for both control and keratoconic groups were recorded in a spreadsheet. Refractive data was recorded for each of the earliest and latest dates of optometry consultations documented as well as an additional two to four consultations in between these dates, when available. Frequencies and quantitative calculations of the mean and standard deviations of changes in BSCVA and cylinder progression over time were undertaken utilising a combination of Excel and SPSS, Version 24, IBM, USA.

## Results

### Demographic data

Data was collected for 952 eyes of 476 control subjects without corneal ectasias or prior refractive surgery and 65 eyes of 37 keratoconic patients that had undergone CXL (refer to S1 File “Minimal Data Set” for raw data). Refractive history for the keratoconic subjects was obtained from a total of 32 different referring optometry practices. Ninety-five percent (n = 34) of patients had been diagnosed with bilateral keratoconus by the treating ophthalmologist and the remaining 5% (n = 3) were diagnosed with unilateral disease. Of the 37 keratoconic patients, 29 underwent CXL in both eyes and eight patients underwent CXL in one eye only. Only the eyes that had undergone CXL were included in the study.

The mean age of control subjects at first recorded presentation to the optometrist was 17.8 ± 6.75 years and 18.7 ± 7.88 years in the keratoconic group. Fifty-seven percent (n = 287/476) of the control subjects were female compared to 42% (n = 16/37) in the keratoconic group. Twenty-four percent of control subjects (n = 116/476) had ocular conditions with dry eye and/or blepharitis the most common (13.3%), and 8.1% (n = 39/476) had systemic disease, with diabetes mellitus (2%), atopic disease (2%) and hypertension (1.4%) the most common. Fourteen percent (n = 5) of keratoconic patients had co-morbid ocular disease, with allergic keratoconjunctivitis the most common (8%), and 22% had co-morbid systemic disease, with atopic disease the most common (18%).

### Patterns in refractive error

The mean spherical equivalent (S.E.) and cylinder power at first presentation to the optometrist, mean rate of change in S.E. and cylinder power per year, and mean difference between subjective and automated refractions for the control group and keratoconic group are summarised in Table 1. While all eyes had recorded subjective refractions, automated refractions were recorded for 54% of subjects (n = 257, 514 eyes) in the control group and only 16% of patients (n = 6, 12 eyes) in the keratoconic group.

**Table 1:** Comparison of refractive error between control and keratoconic groups.

Parameter	Eye	Control Group (dioptres)	Keratoconic Group (dioptres)
Mean S.E. at first presentation to optometrist *	Right eye	-0.30 ± 2.04	-0.67 ± 1.77
	Left eye	-0.23 ± 1.97	-0.85 ± 2.32
	All eyes	-0.27 ± 2.00	-0.79 ± 2.06
Mean cylinder at first presentation to optometrist *	Right eye	-0.36 ± 0.54	-1.45 ± 1.55
	Left eye	-0.38 ± 0.67	-1.51 ± 1.35
	All eyes	-0.37 ± 0.61	-1.75 ± 1.44
Mean rate of change in S.E. per year *	Right eye	-0.03 ± 0.14	-0.29 ± 0.51
	Left eye	-0.01 ± 0.45	-0.54 ± 0.69
	All eyes	-0.02 ± 0.33	-0.41 ± 0.62
Mean rate of change in cylinder per year *	Right eye	-0.01 ± 0.13	-0.60 ± 0.65
	Left eye	0.001 ± 0.292	-0.46 ± 1.17
	All eyes	-0.005 ±0.227	-0.60 ± 0.61
Mean difference between subjective and automated S.E. *	Right eye	0.25 ± 0.60	0.42 ± 1.58
	Left eye	0.24 ± 0.72	0.35 ± 0.76
	All eyes	0.24 ± 0.66	0.39 ± 1.24
Mean difference between subjective and automated cylinder*	Right eye	0.11 ± 0.40	1.73 ± 3.63
	Left eye	0.10 ± 0.68	0.59 ± 1.19
	All eyes	0.11 ± 0.40	1.11 ± 2.56
Difference in S.E. between right and left eyes		0.32 ± 0.31	1.10 ± 2.81
Difference in cylinder magnitude between right and left eyes		0.22 ± 0.18	1.25 ± 2.57
J0 asymmetry		-0.03 ± 0.25	0.16 ± 0.70
J45 asymmetry		0.14 ± 0.16	-0.16 ± 0.89

N.B. “S.E.” and “Cylinder” refer to subjective refractions, unless otherwise stated.

J0 and J45 asymmetry was assessed by calculating the difference in J0 and J45 between the right and left eyes of each patient.

N.B. All differences in value between control and keratoconic groups marked with an asterix in the above table were statistically significant (p<0.05).

N.B. All data is presented as mean +/- standard deviation (SD).

**Fig 1:**
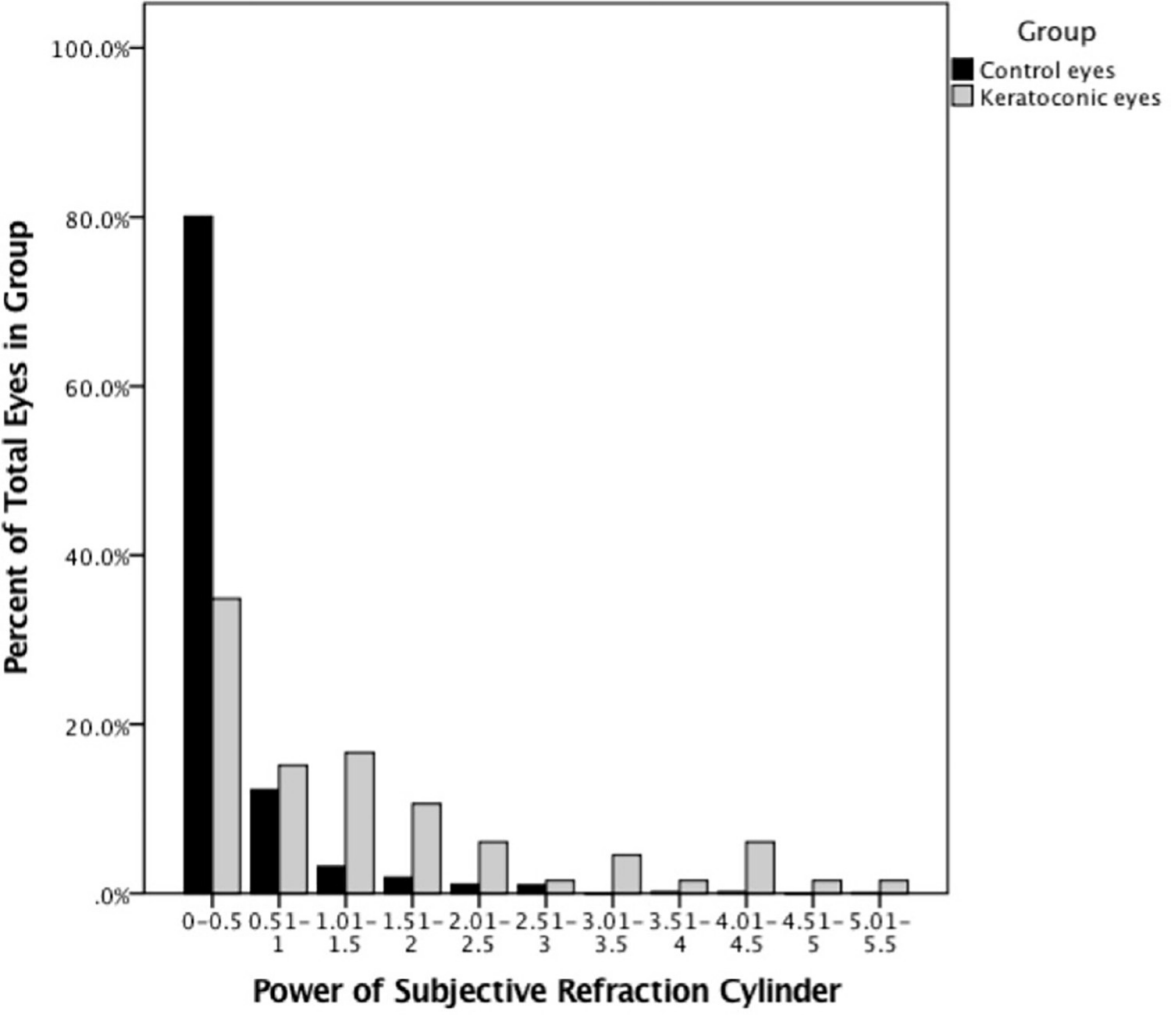
Subjective refraction cylinder at first visit to the optometrist.

As detailed in the table, the mean rate of change in the cylinder power per year, mean rate of change in the S.E. per year, mean difference between subjective and automated S.E. and mean difference between subjective and automated cylindrical power were significantly higher in the keratoconic patients that had undergone CXL compared with the non-keratoconic patients. At first presentation to the optometrist, the mean cylinder as obtained by subjective refraction in the keratoconic eyes also had significantly more toricity than the control group, graphically represented in Fig 1.

**Fig 2:**
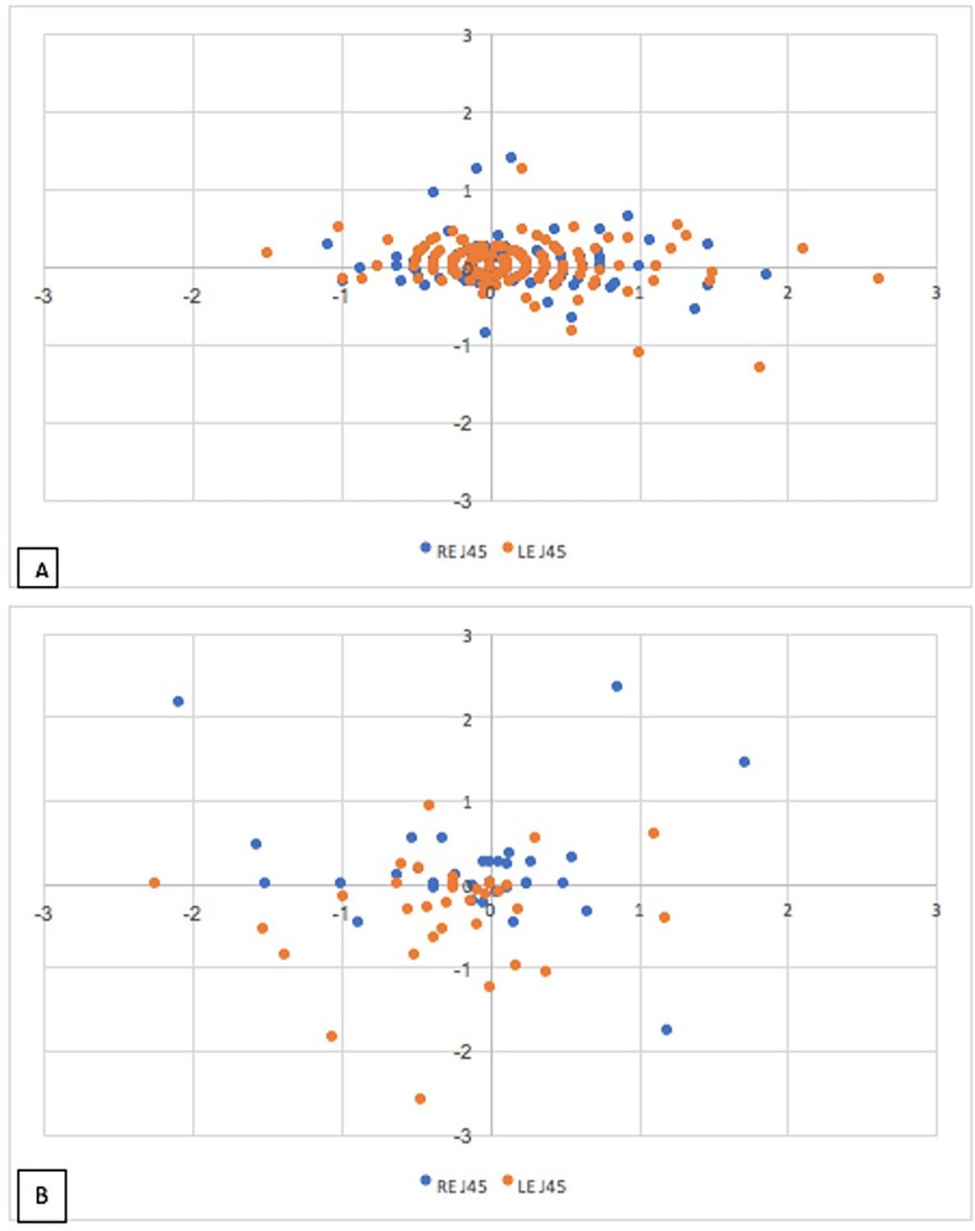
Plot of corneal astigmatism in control (A) and keratoconic Eyes (B) at first visit to the optometrist. Plot of J0 (X axis) vs J45 (Y axis) for control eyes (A) and keratoconic eyes (B) at the first visit. Spread along the X axis signifies the component of astigmatism that is with- (negative direction) or against- (positive direction) the rule. Spread along the Y axis signifies the component of oblique astigmatism. Units are Dioptres.

Utilising the method described by Thibos and colleagues (2001), refractive error can be expressed as spherical equivalent, J0 and J45 power, where J0 power equates to the component of cylinder at 0–180 degrees and J45 power equates to the oblique component of cylinder power. As graphically represented in Fig 2, 40 of the 65 keratoconic eyes (61.2%) had > 1.5 dioptres of astigmatism at the first recorded optometrist visit.

**Fig 3:**
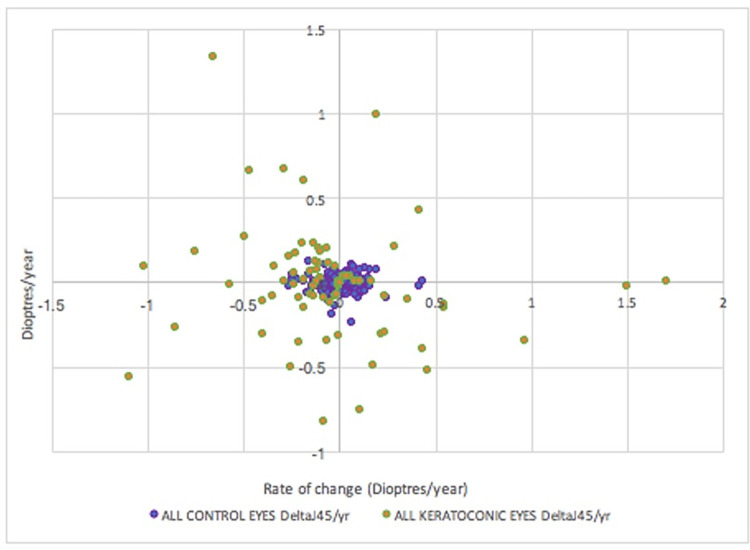
Yearly rate of J0 change (X axis) versus J45 change (Y axis) in control and keratoconic eyes.

In addition to the higher cylinder power observed in the keratoconic eyes, a larger proportion of keratoconic eyes had refractive errors in the oblique rather than vertical or horizontal axis, compared with the control group. As shown in Fig 3, the yearly rate of J0 versus J45 change has a significantly larger spread in the keratoconic eyes compared to control eyes. J0 and J45 asymmetry was assessed by calculating the difference in J0 and J45 between the right and left eyes of each patient. The left eyes’ axes were flipped horizontally in this analysis to respect nasal and temporal correspondence between the eyes. There was greater J0 asymmetry (p<0.001) and J45 asymmetry (p<0.001) in keratoconic subjects when compared to control subjects.

**Fig 4:**
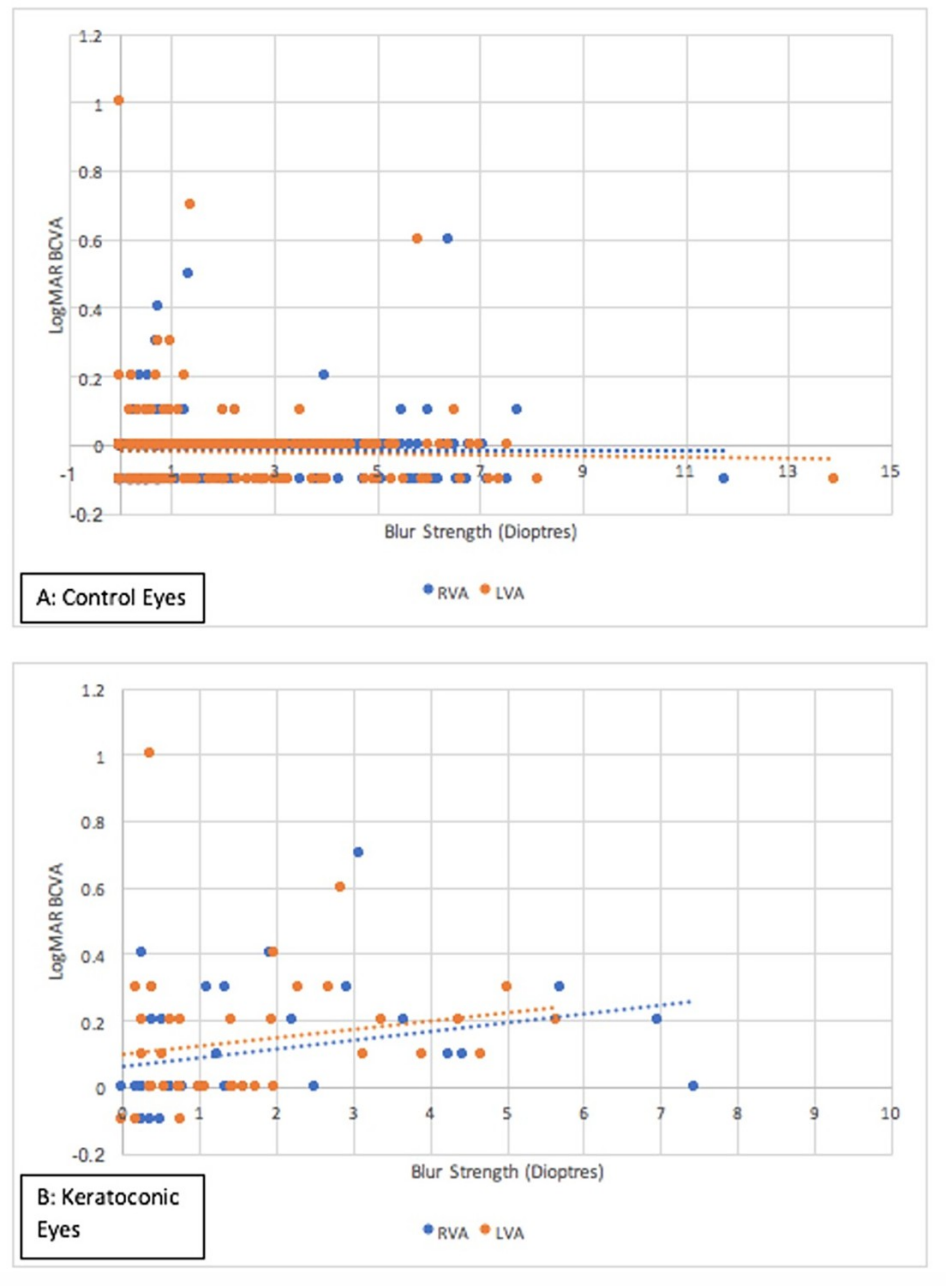
Blur strength versus logMAR BSCVA at first optometrist visit in control (A) and keratoconic eyes (B). The dotted lines represent the lines of best fit for right (blue) and left (orange) eyes.

Thibos and colleagues’ (2001)^12^ methodology of calculating ‘blur strength’ was used to combine the sphere and cylinder defocus power into a single number and plotted against the logMAR BSCVA at first visit to the optometrist for control and keratoconic subjects, shown in Fig 4. The line of best fit for both right and left keratoconic eyes trends upwards whereas this does not occur in the control eyes, suggesting that for the same degree of refractive error, keratoconic eyes have a poorer spectacle-corrected visual acuity than control eyes.

### Trends in BSCVA in keratoconic and control groups

**Fig 5:**
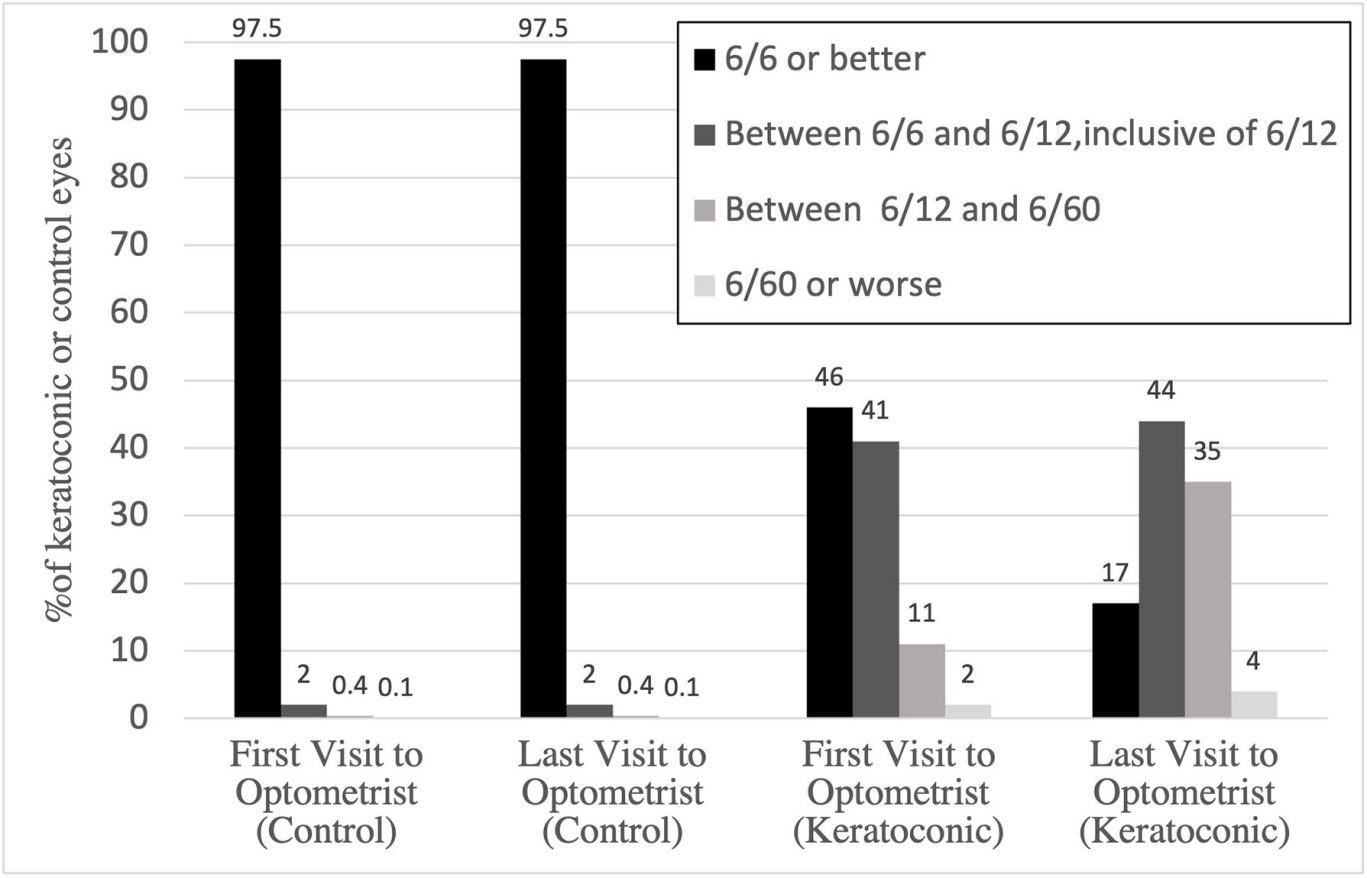
BSCVA at the first and last presentations to optometrists in keratoconic and control eyes.

The BSCVAs at the first and last recorded visits to the optometrist for both control and keratoconic groups are graphically represented in Fig 5. At first visit to the optometrist, 97.5% (n = 78) of control eyes had BCVA equal to or better than 6/6, which remained similar at the last visit to the optometrist. At first visit to the optometrist, 45% (n = 30) of keratoconic patients had visual acuity equal to or better than 6/6, which declined to 37% (n = 25) at the second visit and 25% (n = 17) at the last optometrist visit prior to CXL.

The visual acuity data in Fig 5 demonstrates that BSCVA declined during the period of observation by optometrists prior to ophthalmology referral in the keratoconic patients, however remained relatively stable at first and last optometrist presentations in the control group. Given the difficulties in diagnosing keratoconus in its early stages, patients often present to an ophthalmologist after visual acuity has already declined and disease progression has occurred. Indeed, during the period of observation prior to ophthalmology referral in this study, there was a significant decline in BSCVA in keratoconic patients compared to the control group.

### Time to ophthalmology referral and CXL

**Fig 6:**
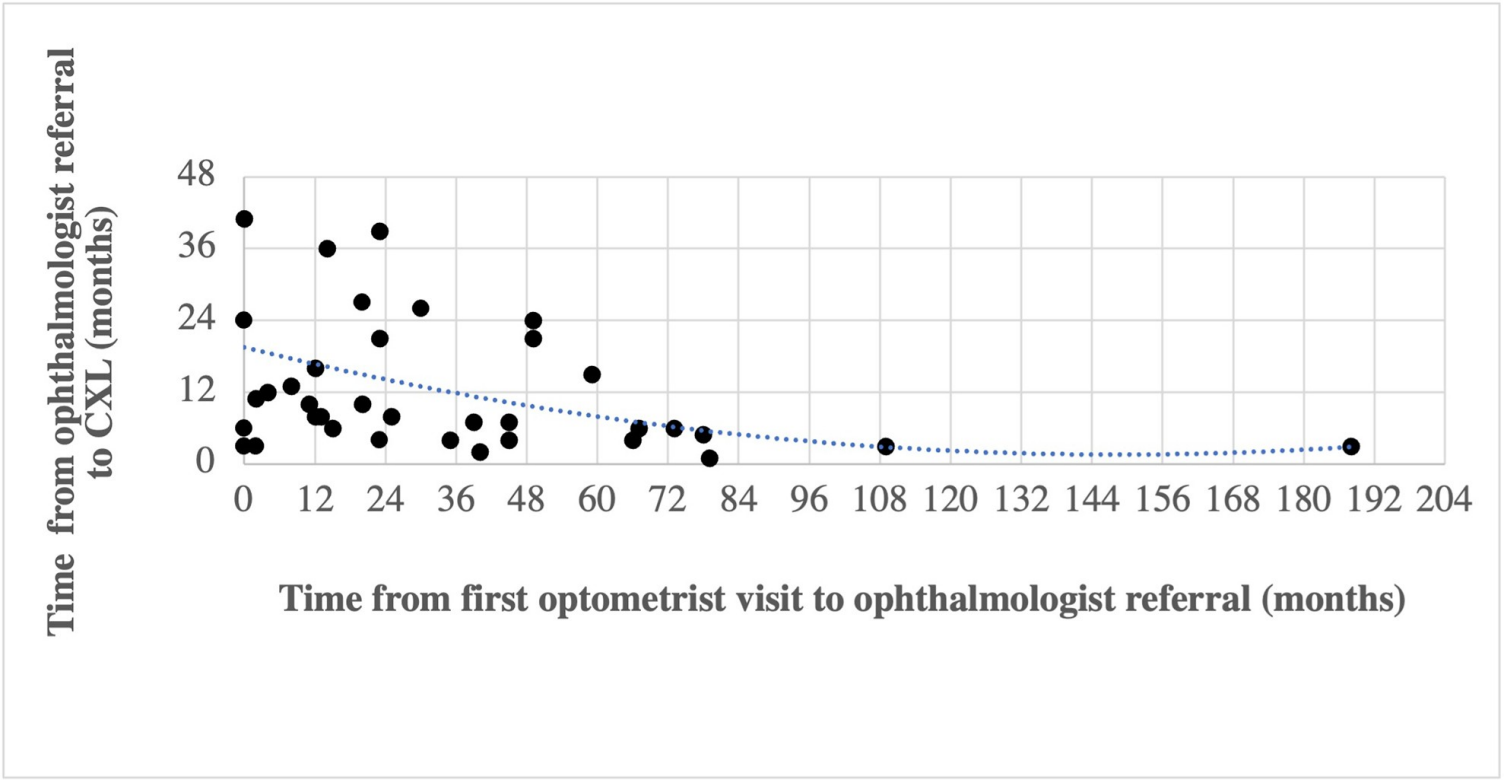
Time from first optometrist visit to ophthalmologist referral and CXL.

In the control group, the total mean duration of follow-up recorded by optometrists was 7.6 ± 2.1 years. In the keratoconic group, the mean duration from the first recorded optometrist visit to ophthalmology referral was 2.9 ± 3.1 years. The time from the first optometrist visit to ophthalmology referral and time from ophthalmology referral to CXL for the keratoconic patients is shown in Fig 6. The mean time period from when patients were referred for ophthalmology review by the optometrist to CXL (calculated as the date of CXL of the first eye in patients with bilateral keratoconus) was 1.0 ± 0.9 years.

As graphically represented in Fig 6, 33% of patients were referred to ophthalmologists within 12 months of their first optometrist visit and 60% were referred within 24 months. Seventy-three percent of patients underwent CXL within 12 months of referral to an ophthalmologist. On average, a longer duration of observation by the optometrist prior to ophthalmology referral was associated with a shorter period of observation by the treating ophthalmologist, as demonstrated by the polynomial trendline. Based on this data, optometrists will have followed up 50% of their patients for three years prior to ophthalmology referral whilst ophthalmologists will have crosslinked 50% of keratoconic patients within one year of referral.

Notably, the timing of the first recorded presentation to an optometrist compared with the timing of diagnostic suspicion and subsequent referral to an ophthalmologist varied between keratoconic patients, with some patients having had keratometry at their optometrists and immediate referral to an ophthalmologist whilst others were followed up for a longer period of time before suspicion was raised.

**Fig 7:**
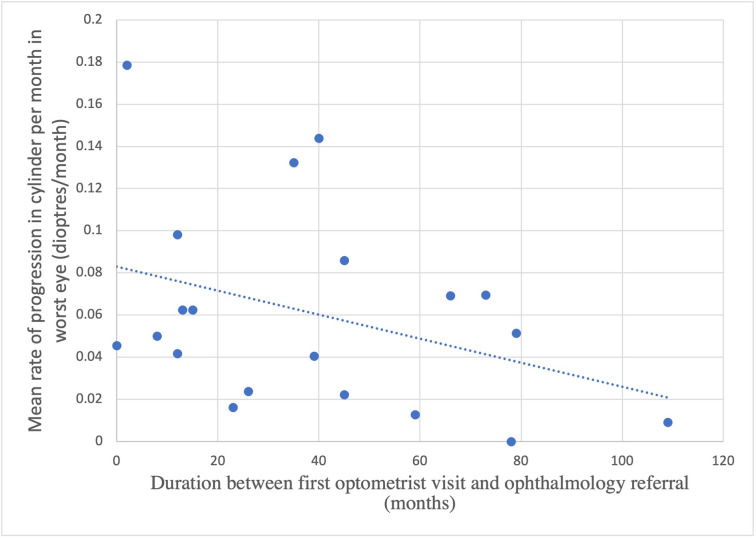
Duration of observation prior to ophthalmologist referral compared with progression in subjective refraction cylinder power per month in keratoconic eyes. NB. Results are shown for the keratoconic eye with the greater progression in cylinder power per month for each patient to avoid duplication of results, with similar trends observed for the corresponding eyes.

Fig 7 demonstrates that keratoconic eyes which progressed more slowly, as measured by progression in subjective refraction cylinder power per month were more likely to experience a longer delay in referral to an ophthalmologist. This can be compared to the control group’s almost negligible progression in cylindrical power of -0.005 ±0.227 per month. Similarly, it was found that a better BSCVA at first optometry visit and a slower rate of loss in BSCVA per month were associated with longer delays in ophthalmology referral.

### Clinical relevance of the timing of ophthalmology referral

The mean rate of loss in BSCVA per year in keratoconic patients during their recorded optometrist visits prior to CXL was 0.12 ± 0.18 logMAR per year, which equates to 6 ETDRS letters lost per year. Given that the mean duration from the first recorded optometrist visit to ophthalmology referral was 2.9 ± 3.1 years, over this average delay of 2.9 years, there would be a mean decline in BSCVA of approximately 0.35 logMAR, equivalent to 17 ETDRS letters.

## Discussion

This study documents multiple refractive parameters including the mean cylindrical power at first optometry visit, mean rate of change in cylinder power per year, mean rate of change in the S.E. per year, mean difference between subjective and automated S.E. and mean difference between subjective and automated cylindrical power, which were significantly higher in the keratoconic patients that had undergone CXL compared with non-keratoconic patients. It also documents the relationship between blur strength, in which sphere and cylinder defocus power were combined into a single number as per Thibos and colleagues’ (2001) methodology, and BSCVA, indicating that for the same degree of refractive error, keratoconic eyes have a poorer BSCVA than control eyes [28]. This observed limited correction of visual acuity with spectacles in keratoconic patients is consistent with the presence of irregular astigmatism and higher order aberrations demonstrated in clinical practice. Notably, to the authors’ knowledge, this is the first study to document refractive change over time in keratoconic patients, with data dating from prior to ophthalmology referral and diagnosis, and provide a comparison with non-keratoconic subjects.

The data demonstrates that keratoconic patients that were eventually determined by a corneal specialist to be likely to benefit from CXL are usually referred to an ophthalmologist earlier when the BSCVA is poorer at initial presentation to the optometrist, if there is a greater rate of decline in visual acuity and decline in S.E. and cylinder progression over time. This suggests that as optometrists detect notable changes in visual acuity and refractive error, they increasingly consider ophthalmology referral. Patients with less significant rates of decline, however, are more likely to be missed as potential keratoconic patients when reviewed by their optometrists. Without access to dedicated imaging modalities such as tomography and topography and no established screening tool for keratoconus based on changes in visual acuity or refraction, optometrists are limited in their ability to determine whether deterioration in BSCVA is secondary to patient and testing factor variability or an underlying undiagnosed pathology such as keratoconus.

In the keratoconic cohort, the mean duration from the first recorded optometrist visit to ophthalmology referral was 2.9 ± 3.1 years, which correlated with a mean decline in BSCVA of approximately 0.35 logMAR, equivalent to 17 ETDRS letters. Essentially, a patient’s BSCVA could decline from 6/6 to worse than 6/12 prior to being referred to an ophthalmologist for definitive diagnosis and treatment. This demonstrates a role for a suitably sensitive, accurate and specific optometry-based screening tool to help facilitate earlier specialist referral of patients deemed at high-risk of developing vision-threatening, progressive keratoconus.

Limitations of the study include the non-randomised geographical distribution of patients, with control subjects recruited from two optometry practices in Wollongong, whilst keratoconic patients were recruited across three ophthalmic institutions in New South Wales. The age distribution and follow-up period of patients, however, was similar between the control and keratoconic groups. The small population size of the keratoconic group may have contributed to more kurtotic results compared with the control group, which does raise the potential for a skew in the mean values reported, although the differences between the groups remains significant. Also of relevance, whilst the vast majority of optometry practices contacted did not have access to a topography, a small proportion did, which may have facilitated earlier referral independent of the deterioration in BSCVA and cylinder progression. The data, however, is consistent in demonstrating that there remains a time lag between a keratoconic patient’s first visit to their optometrist and the point at which they are referred to a corneal specialist for investigation, and this time period is also associated with a significant decline in BSCVA.

It is also relevant to note that the study covers a surveillance period between 2016–2017, which is some time ago and prior to the COVID pandemic. Since then, optical coherence tomography (OCT) has become available, including epithelial mapping [10, 29]. Keratoconus enlargement, widely considered as more than one dioptre in the anterior curvature of non-apical corneal areas, is also cited in recent literature as a useful tool, but is still not in common usage in clinical practice [30]. Despite the time that has passed since the study period, Scheimpflug imaging remains the most commonly utilised diagnostic tool [31–33]. Guidelines for CXL have also not changed significantly since the study period, with consensus for keratoconus progression still largely utilising the ABCD progression and the criteria of 1–1.5D increase in Kmax over one year [9, 24]. Although more optometrists are purchasing corneal topographers primarily for the fitting of complex contact lens designs and especially for orthokeratology contact lens fittings, this instrumentation is not widely available in optometric practice. Moreover, topographers are rarely used as a screening tool for the general population in whom keratoconus has not yet been identified and is still in the early stages, hence the rationale behind collecting and analysing refractive data in this study. A recent study of optometry referral patterns in New Zealand reported that ownership of a corneal imaging unit did not alter referral patterns for keratoconus [12]. This is consistent with the authors’ opinion that refractive data and BSCVA remain more commonly utilised tools by optometrists in early keratoconus, and thus the most potentially useful for early diagnosis given the significant refractive differences over time between control and keratoconic groups identified in this study.

The inclusion of CXL for keratoconus progression in Australia’s national healthcare rebate system, Medicare, in 2018 is another interesting discussion point. This may have created increased awareness of the urgency of referring patients with progressive keratoconus compared with 2016, however it is the authors’ view that significant referral delay still exists. Although the Medicare rebate most likely improved access to CXL services in the private system, it is unlikely to have influenced referral delays from optometrists. This study only included patients that had opted to proceed with CXL in the private system, despite the lack of rebates at the time, and so may have introduced bias in observing the more affluent keratoconic population. It is the authors’ opinion that the real referral delay is likely to be greater than reported in this study.

It has been shown that there is usually clear topographic evidence of keratoconus for some time prior to recordable loss of BSCVA [31], thus providing an opportunity to reduce the referral delay that currently exists with an adequate optometry-based screening tool. Optometrists are faced with screening for a corneal disease that is uncommon, and for which specialised equipment is necessary for diagnosis but often unavailable in optometry practices. Ophthalmologists are referred a small number of relatively high-risk patients and are faced with a different problem–whether the keratoconus is progressive to the point of threatening vision in the future and therefore requiring invasive management. Crosslinked patients are too often left with significant, permanent vision loss because of a delay in diagnosis and treatment. It is the authors’ opinion that we should be striving to reduce this delay. While there is significant research and various tools available to aid in confirming a diagnosis and keratoconic progression, optometrists most frequently utilise refractive data and BSCVA for screening. A 2015 survey reported that 45% of Australian optometrists had a topographic unit in their practice at the time, and optometric uptake of ocular topography imaging technology has increased in the past seven years since the study period [12]. The data supports the notion that subclinical and early keratoconus is often missed and suggests that the current tools available to optometrists for detecting early keratoconus are suboptimal by documenting a delay in specialist referral of patients at risk of keratoconus. Noting the significant differences in refractive error parameters and refractive change over time between non-keratoconic subjects and keratoconic patients in this study, it suggests a potential role for refractive parameters to be included in such a screening tool. The refractive changes documented over time prior to diagnosis and ophthalmology referral in keratoconic subjects also provides useful information in the clinical gestalt for both optometrists and ophthalmologists in monitoring and diagnosing patients with subclinical keratoconus.

## Conclusions

The lack of a universal classification and grading system makes the diagnosis of keratoconus challenging, particularly in cases of subclinical or *forme fruste* keratoconus. Extended periods of observation of keratoconic patients prior to specialist referral are associated with significant decline in BSCVA which is currently often unavoidable, given the limited availability of specialist diagnostic equipment in optometry practices. This study documents patterns in refractive error and visual acuity progression in keratoconic patients that may assist optometrists in monitoring patients with early disease and detecting those that would benefit from specialist referral. It also highlights the urgent need for a keratoconic screening tool that would raise suspicion of a diagnosis of keratoconus earlier than current optometric clinical tools and acumen provide.

## Supporting information

S1 File Minimal data set. (XLSX)

S2 File Ethics committee approval. (PDF)
