# Supplementary material for: Patterns in refractive error and treatment delay in keratoconus–An Australian study
Source: PLoS One. 2024 Jan 11;19(1):e0297268. doi: 10.1371/journal.pone.0297268 (PMC10783750; doi:10.1371/journal.pone.0297268)
Supplement: S2 File — (PDF) [file pone.0297268.s002.pdf]

Health
South Eastern Sydney
Local Health District

HUMAN RESEARCH ETHICS COMMITTEE
Room G71 East Wing
Edmund Blacket Building
Prince of Wales Hospital
RANDWICK NSW 2031
Tel: 02 9382 3587 Fax: 02 9382 2813
SESLHD-RSO@health.nsw.gov.au
www.seslhd.health.nsw.gov.au/POWH/researchsupport

01 September 2017

Dr Yves Kerdaron
Attn: Dr Samantha Bobba
Dept Ophthalmology
Sydney Eye Hospital
Macquarie St
SYDNEY NSW 2000

Dear Dr Kerdaron

HREC ref: 17/137 (LNR/17/POWH/289)
Project: Analysing Refractive Error in Early Keratoconus and its Potential as a Screening Tool

Thank you for submitting the above Low/Negligible Risk (LNR) Application for review by the Human Research Ethics Committee (HREC). Based on the information you have provided and in accordance with the NHMRC guidelines [National Statement 2007 – Section 5 Institutional Responsibilities and "*When does quality assurance in health care require independent ethical review?*" (2003)], this project has been assessed as low risk and is therefore exempt from full HREC review.

Thank you for your correspondence dated **20 August 2017** responding to queries. The project was first considered by the LNR Committee on **09 May 2017**.

I am pleased to advise that ethics approval has been granted for this project to be conducted at the following site(s):

- Sydney Eye Hospital
- ANG Optical (Eyestore, Warilla)
- Hue Optix Pty Ltd (Eyecare Plus, Wollongong)

The following documentation has been approved:

- LNR Form submission AU/6/0C7D211 dated 27 April 2017
- Participant Information Sheet & Consent Form v2 dated 29 May 2017
- Data collection sheet, not dated

<u>Conditions of approval</u>

1. This approval is valid for 5 years from the date of this letter.
2. Annual reports must be provided on the anniversary of approval.
3. A final report must be provided at the completion of the project.
4. Proposed changes to the research protocol, conduct of the research, or length of approval will be provided to the Committee.
5. The Principal Investigator will immediately report matters which might warrant review of ethical approval, including unforeseen events which might affect the ethical acceptability of the project and any complaints made by study participants.

For NSW Public Health sites only: You are reminded that this letter constitutes ethics approval only. You must not commence this research project until you have submitted your Site Specific Assessment (SSA) to the Research Governance Officer of the appropriate institution and have received a letter of authorisation from them.

Should you have any queries, please contact the Research Support Office on (02) 9382 3587. The HREC Terms of Reference, Standard Operating Procedures, membership and standard forms are available from the Research Support Office website: http://www.seslhd.health.nsw.gov.au/POWH/researchsupport/default.asp.

Please quote **HREC ref no: 17/137** in all correspondence.

We wish you every success in your research.

Yours sincerely

Andrew Bohlken
Executive Officer, Human Research Ethics Committee

This HREC is constituted and operates in accordance with the National Health and Medical Research Council's (NHMRC) *National Statement on Ethical Conduct in Human Research (2007)*, NHMRC and Universities Australia *Australian Code for the Responsible Conduct of Research (2007)* and the *CPMP/ICH Note for Guidance on Good Clinical Practice.*
